# Circulating tumor cells capture disease evolution in advanced prostate cancer

**DOI:** 10.1186/s12967-017-1138-3

**Published:** 2017-02-23

**Authors:** Justin Lack, Marc Gillard, Maggie Cam, Gladell P. Paner, David J. VanderWeele

**Affiliations:** 1 Center for Cancer Research Collaborative Bioinformatics Resource, Center for Cancer Research, National Cancer Institute, Bethesda, MD 20892 USA; 2 Department of Surgery, University of Chicago, Chicago, IL 60615 USA; 3 Department of Pathology, University of Chicago, Chicago, IL 60615 USA; 4 Laboratory for Genitourinary Pathogenesis, Center for Cancer Research, National Cancer Institute, 37 Convent Drive, Rm 1066A, Bethesda, MD 20892 USA; 5 Department of Medicine, University of Chicago, Chicago, IL 60615 USA

**Keywords:** Circulating tumor cells, Castrate resistant prostate cancer, Tumor evolution, Neuroendocrine prostate cancer

## Abstract

**Background:**

Genetic analysis of advanced cancer is limited by availability of representative tissue. Biopsies of prostate cancer metastasized to bone are invasive with low quantity of tumor tissue. The prostate cancer genome is dynamic, however, with temporal heterogeneity requiring repeated evaluation as the disease evolves. Circulating tumor cells (CTCs) offer an alternative, “liquid biopsy”, though single CTC sequencing efforts are laborious with high failure rates.

**Methods:**

We performed exome sequencing of matched treatment-naïve tumor tissue, castrate resistant tumor tissue, and pooled CTC samples, and compared mutations identified in each.

**Results:**

Thirty-seven percent of CTC mutations were private to CTCs, one mutation was shared with treatment-naïve disease alone, and 62% of mutations were shared with castrate-resistant disease, either alone or with treatment-naïve disease. An acquired nonsense mutation in the Retinoblastoma gene, which is associated with progression to small cell cancer, was identified in castrate resistant and CTC samples, but not treatment-naïve disease. This timecourse correlated with the tumor acquiring neuroendocrine features and a change to neuroendocrine-specific therapy.

**Conclusions:**

These data support the use of pooled CTCs to facilitate the genetic analysis of late stage prostate cancer.

**Electronic supplementary material:**

The online version of this article (doi:10.1186/s12967-017-1138-3) contains supplementary material, which is available to authorized users.

## Background

Prostate cancer biology is initially dominated by activity of the androgen receptor (AR), and androgen deprivation therapy (ADT) is the backbone of therapy for those with metastatic prostate cancer. Castrate resistance is nearly universal, however, requiring additional management decisions. The heterogeneity of primary prostate cancer is well established [1–3], and polyclonal seeding of metastatic sites, and metastasis-to-metastasis spread, appear to be possible, if not common [4]. Moreover, the prostate cancer genome is dynamic, with different clones dominating over time in response to different lines of therapy [5, 6]. As the number of approved agents that prolong survival increases, the dynamic clonal nature of this disease is likely to become a greater issue. For example, there appears to be a new entity, intermediate between conventional adenocarcinoma and classic neuroendocrine disease, that arises with resistance to newer AR-targeted agents [7].

Circulating tumor cells (CTCs) and cell free DNA (cfDNA) are emerging as non-invasive alternative means to interrogate the genetics of late stage disease rather than invasive and/or risky biopsy of metastatic disease. They are complimentary methods, each with advantages and disadvantages. The dilute tumor fraction of cfDNA in most patients requires very high sequencing coverage to detect mutations, typically limiting analysis to a small number of targeted regions [5]. On the other hand, amplifying DNA from single circulating tumor cells is inconsistent and low yield. Successful efforts from individual cases have sequenced four of 99 collected CTCs, or pooled the results from 19 separately sequenced CTCs [8, 9]. We present here whole exome sequencing of pooled CTCs, with matched treatment-naïve and castrate resistant tissue, demonstrating identification in pooled CTCs of clinically relevant mutations acquired late in the course of disease.

## Methods

### Patient recruitment and patient samples

The patient provided written informed consent and was enrolled in the “Prostate Cancer Sample Collection” protocol of the University of Chicago Medical Center, which was approved by the University of Chicago ethics committee (approval reference number 13-1295). Biopsy specimens were processed per clinical protocols including hematoxylin and eosin stain and immunohistochemical analysis of PSA expression. Circulating tumor cells were collected as previously described [10]. In short, mononuclear cells were enriched from 15 ml peripheral blood and stained with an Alexa-488 conjugated EpCAM antibody (Biolegend, 1:100) and a QDot800 conjugated CD45 antibody (Invitrogen, 1:100). CTCs were isolated by FACS-sorting EpCAM+/CD45− cells on a MoFlo XDP flow-sorting machine. One thousand WBCs were isolated by FACS-sorting EpCAM−/CD45+ cells as representative of germline DNA.

### Whole genome amplification and exome sequencing

Isolated EpCAM+/CD45− CTCs were divided into three equal samples of 500 cells each and independently subjected to whole genome amplification (WGA) through multidisplacement amplification using REPLI-G (Qiagen), following the manufacturer’s instructions, as were EpCAM−/CD45+ WBCs representative of germline. The quality of amplification was evaluated through PCR amplification of eight targets [11] to evaluate uniform amplification across multiple chromosomes, and length of amplification product. Primers used are listed in Additional file 1. Exome sequencing libraries were prepared using NEB Next Ultra (New England Biolabs) kit following the manufacturer’s instructions. Sequencing was performed on a HiSeq 2000 (Illumina).

### Sequencing analysis

Illumina sequencing data was mapped to the GRCh37 version of the human reference genome using BWA–MEM [12] and further processed following the GATK Best Practices [13]. Somatic variants were called for each tumor or CTC sample in a paired manner using MuTect [14] in high confidence mode. Potential germline variants were removed by excluding positions with germline coverage <20×, variant reads in the germline, or variants present in dbSNP, and remaining variants were enriched for highest confidence by including only variants in the exome and with 5 or more supporting reads comprising 10% or more of all reads. Somatic mutations passing all filters (listed in Additional file 2) in a given sample were then examined for the presence/absence of supporting reads in other samples using samtools mpileup [15, 16]. Mutations were visually verified by examining supporting reads using Alview [17], and all somatic mutations were annotated using AVIA [18].

## Results

**Fig. 1:**
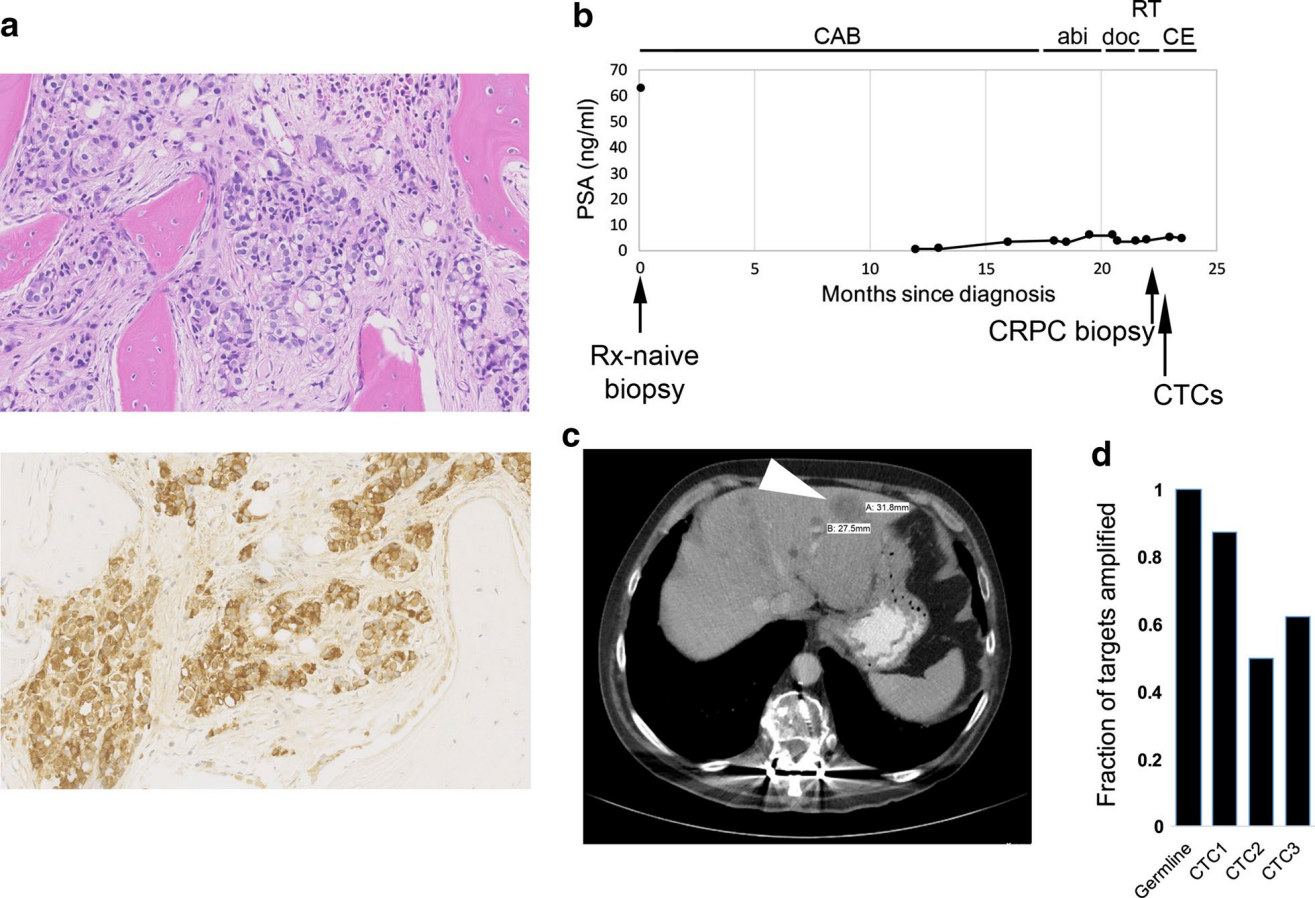
Clinical timecourse of disease. **a** Hematoxylin-Eosin stain (*top*) and immunohistochemical analysis of PSA protein (*bottom*) of treatment-naive bone biopsy diagnosing metastatic prostate cancer. **b** Clinical timecourse indicating PSA levels, timing of biopsies, timing of the single CTC collection ~3 weeks after CRPC biopsy, which was divided into three pools, and timing of therapies. *CAB* combined androgen blockade, *abi* abiraterone, *doc* docetaxel, *RT* palliative radiation therapy, *CE* carboplatin and etoposide. **c** CT image of liver metastasis that underwent biopsy (*white arrowhead*). **d** Fraction of PCR targets that were amplified from each whole genome amplification product

A 70 year old patient was diagnosed with metastatic prostate cancer, confirmed with biopsy of a bone metastasis (Fig. 1a). He underwent androgen deprivation therapy but eventually developed castrate resistant prostate cancer (CRPC). Additional conventional therapies yielded short-lived responses and subsequent progression of disease (Fig. 1b). A liver biopsy performed 22 months after the initial diagnosis confirmed metastatic prostate cancer (Fig. 1c), but with neuroendocrine and small cell features, which was not appreciated on the initial biopsy. Given the change in dominant histology he started treatment with carboplatin and etoposide, but after two cycles he entered hospice care.

To evaluate the extent to which the current disease biology can be discovered through CTCs, CTCs were collected from 15 ml of peripheral blood within a month of the liver biopsy. CTCs were enriched by FACS-sorting EpCAM+/CD45− cells as previously described [10] and divided into three equal fractions. Multidisplacement amplification was used to amplify the entire genome of each fraction independently. To evaluate for loss of coverage due to lack of amplification and for length of amplified fragments, the amplified CTC genomes were evaluated with endpoint PCR for eight targets of varying lengths across six different chromosomes (Fig. 1d). More targets were able to be amplified from one CTC pool (CTC1) than the other two pools (CTC2, CTC3). All three samples were used for exome sequencing.

**Fig. 2:**
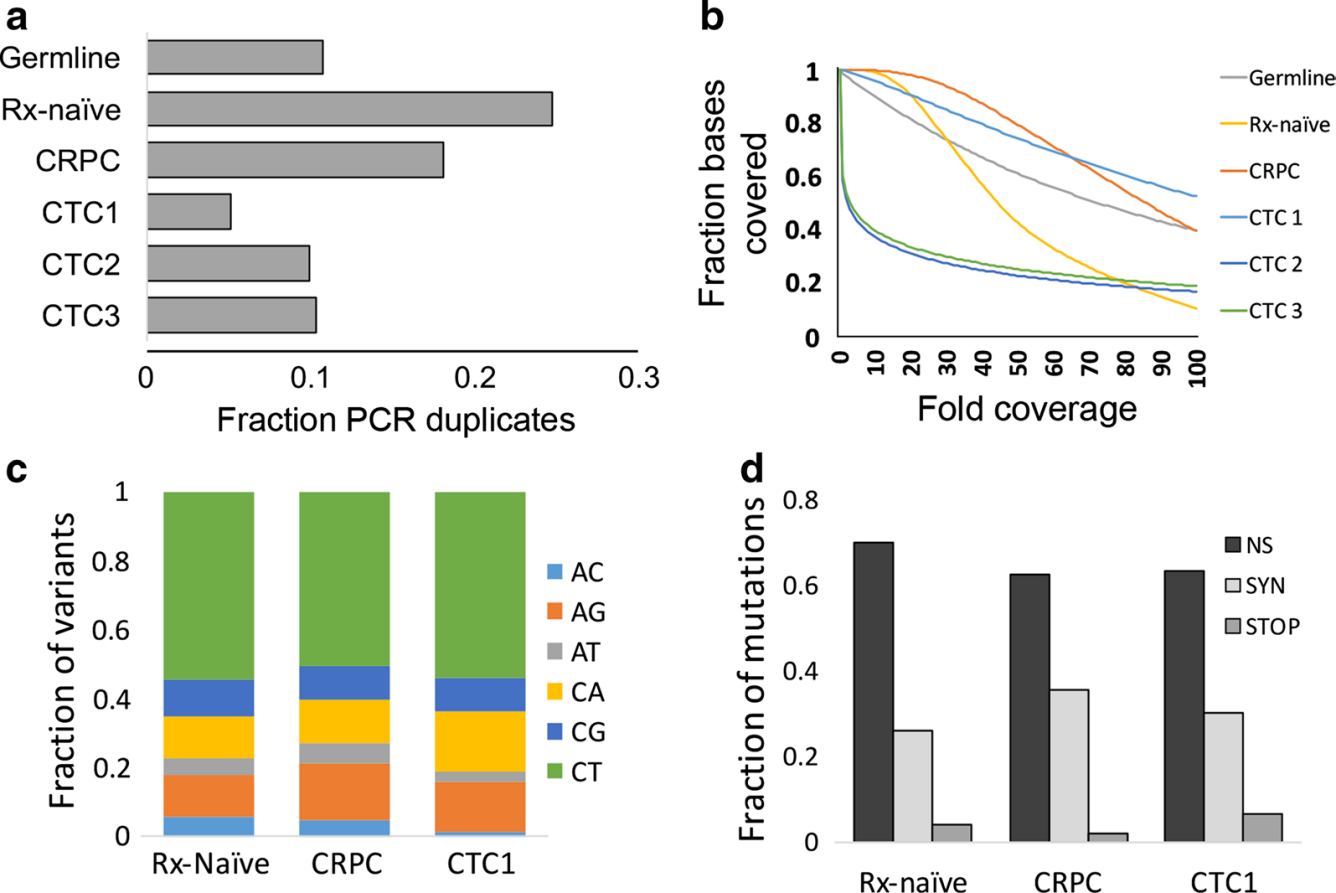
Similar sequencing library characteristics from tissue and one of three CTC pool sequencing libraries. **a** Fraction of sequencing libraries consisting of PCR duplicates. **b** Coverage plots for germline, treatment-naïve (Rx-naïve) tissue, advanced cancer (CRPC) tissue, and three CTC pool sequencing libraries. **c** Transitions and transversions and **d** mutation types among the variants identified in the two tissue and CTC1 sequencing library

Whole exome sequencing was performed on a germline sample and five tumor samples: initial diagnostic, treatment-naive biopsy of an ischial metastasis; liver biopsy of CRPC; and three samples of pooled CTCs divided from a single CTC collection. The fractions of PCR duplicates were all 0.25 or less (Fig. 2a). Adequate coverage was obtained for the germline sample, the tissue samples, and CTC1, samples that were predicted to perform well by initial evaluation, with coverage of over 98% of the genome, median depths of 44–106×, and 10× coverage of 90% or more of the exome (Fig. 2b). The lower quality CTC samples covered just 37 and 40% of the exome at 10×. Due to their poor quality they were left out of downstream analysis. Mutation patterns were consistent in the treatment-naïve, CRPC, and CTC sample, including nucleotide substitutions (Fig. 2c) and type of mutation (Fig. 2d).

**Fig. 3:**
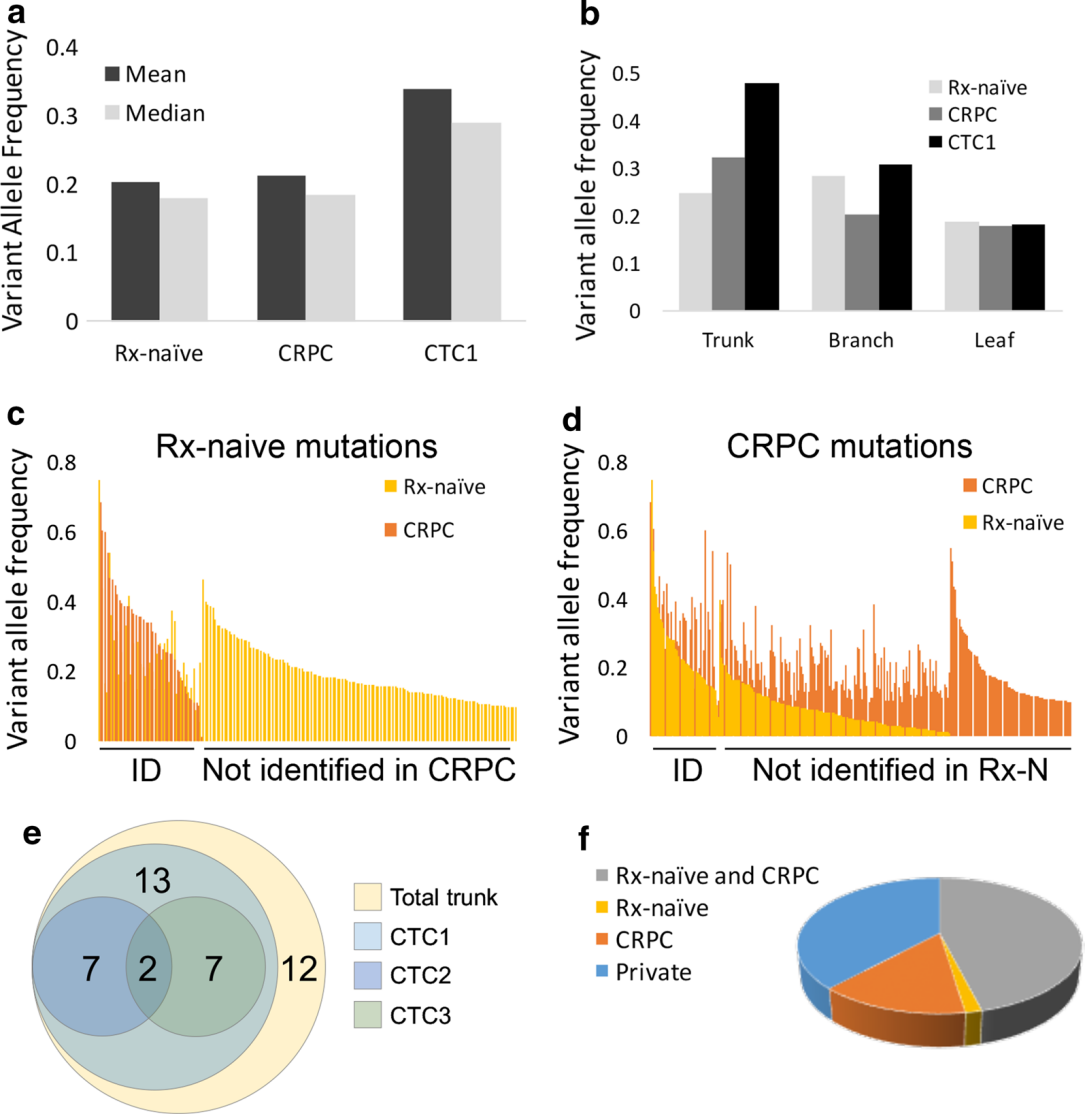
Circulating tumor cells pool captures majority of trunk mutations and additional mutations from metastatic disease. **a** Variant allele frequencies for treatment-naïve, CRPC, and CTC1 mutations. **b** Average variant allele frequencies of trunk, branch, and leaf mutations in two tissue samples and one CTC sample. **c** Allele fraction in CRPC tissue of mutations identified in treatment-naïve tissue, and **d** vice versa. On the left are mutations identified in both samples. On the right are mutations identified in only sample. In **d**, many of the mutations not identified independently in treatment-naïve tissue had evidence they were present at low allele frequency. **e** Number of trunk mutations identified in three pooled CTC sequencing libraries. **f** Fraction of CTC mutations shared with other tissue samples

The allele frequency was comparable for mutations in the tissue specimens, but higher in the CTCs (Fig. 3a). Moreover, while trunk mutations (those found in both treatment-naïve and CRPC tissue samples) were near 50% allele frequency, suggesting close to 100% pure CTCs all with one allele mutated, branch (shared with one tissue sample) and leaf (found only in CTCs) mutations were at lower allele frequency, indicating genetic heterogeneity among CTCs (Fig. 3b).

There was considerable genetic heterogeneity among tumor tissue samples, with just 8% of mutations being trunk mutations, and 25% being found in two samples. Thirty-six percent of mutations from CRPC were not initially identified in the treatment-naive sample but had at least two reads supporting their presence, indicating enrichment in the CRPC tissue of subclonal populations from the treatment-naive tissue. The reverse—mutations identified in the treatment-naive sample with low frequency supporting reads in CRPC—was not identified (Fig. 3c, d). The pooled CTCs identified 71% of mutations shared by treatment-naïve and CRPC tissue samples, suggesting most mutations at high allele frequency (early mutations) were represented in the CTCs (Fig. 3e). Examining the two low quality CTC pools, each pool identified 22% of trunk mutations, consistent with their 10x coverage being 37–40% of the genome. All the trunk mutations identified in the low quality pools were also identified in the high quality pool, suggesting consistency in the mutations able to be identified by CTCs.

The CTCs had the highest fraction of mutations shared with at least one other sample, at 64% (Fig. 3f). Fifty percent were trunk mutations, shared with both tissue samples, and an additional 14% were shared with the CRPC sample alone. A single mutation was shared between CTCs and treatment-naïve tissue and not found in CRPC tissue. Though the other two CTC samples had poor exome coverage and were not of sufficient quality to identify mutations a priori, 33% of mutations identified in the high quality CTC sample were also identified in one of the low quality CTC samples.

**Fig. 4:**
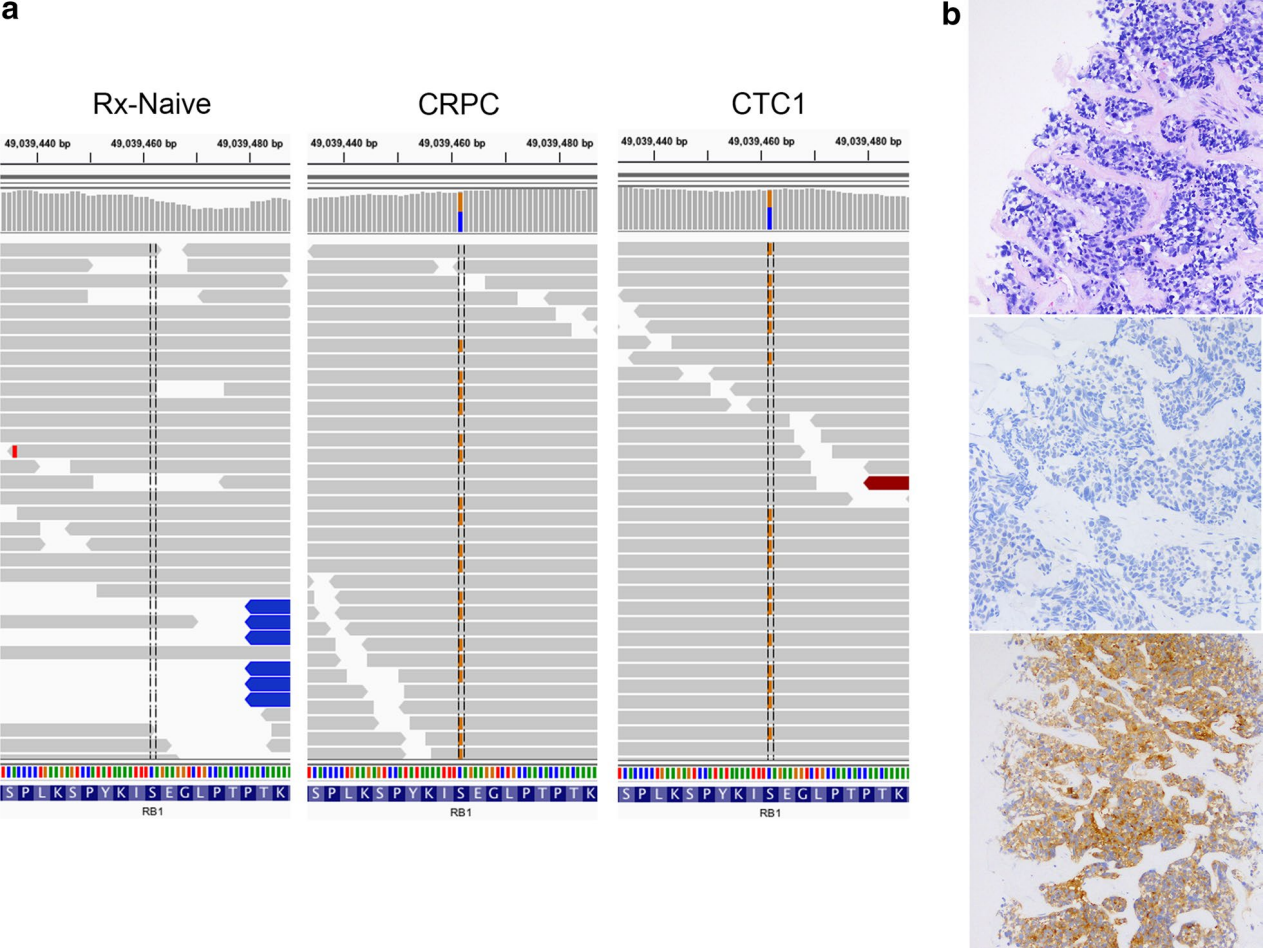
Identification of RB1 nonsense mutation in CRPC tissue and CTC samples. **a** Integrative Genomics Viewer [28] images of the S816X mutation identified in CRPC and CTC1, but not treatment-naïve sample. The *brown bars* in the *middle panels* represent the mutated nucleotide and its position within the sequencing reads. The histograms in the *upper panels* summarize the fraction of reference (*gray* or *blue*) or mutated (*brown*) reads. **b** Hematoxylin-Eosin stain (*top*) and immunohistochemical analysis of PSA (*middle*) or synaptophysin protein (*bottom*) of the CRPC liver biopsy

One of the mutations shared between the CRPC tissue sample and CTCs was a premature stop codon in the Retinoblastoma (RB1) gene (Fig. 4a), which is associated with isolated bilateral retinoblastoma and meningioma [19]. The appearance of this RB1 mutation coincided with loss of adenocarcinoma features, including PSA expression (compare Fig. 4b, top and middle panels, to Fig. 1a), and gain of neuroendocrine features in the tumor, which was confirmed by synaptophysin expression (Fig. 4b, bottom panel). This phenotypic change toward therapy-emergent neuroendocrine prostate cancer prompted a change in management strategy away from standard of care for CRPC.

## Discussion

The prostate cancer genome is heterogeneous, both between and within the multiple foci characteristic of primary disease. The clonal architecture of advanced disease is dynamic, with new clones gaining dominance in response to new therapies. Combined, these necessitate repeated genomic or molecular assessments over the course of disease in order to have a complete, current understanding of a patient’s personalized disease.

Circulating tumor cells offer a non-invasive mechanism to repeatedly evaluate the shifting dynamics of disease. This has demonstrated clinically meaningful evaluations of specific alterations [20, 21]. However, there have been few direct correlations between treatment-naïve tissue, CTCs, and contemporaneous CRPC tissue, and wider scale genomic evaluations are still in early stages. Lohr et al. used whole genome sequencing to evaluate quality of amplified DNA followed by exome sequencing of 19 individual CTCs to demonstrate late divergence of CTCs and a previously resected lymph node [8]. Jiang et al. used laser capture microdissection to capture and evaluate 99 individual CTCs collected over five blood collections, identifying four individual CTCs with high quality DNA and eight with moderate quality [9]. The CTCs in that study identified 15% of trunk SNVs, with supporting reads for an additional 14% of reads.

Using our pooled CTC strategy, we generated successful sequencing libraries from 33% of samples. The sequencing demonstrated high correlation with tissue samples, confirming the biological relevance of the CTC exomes, identifying 71% of trunk mutations, along with additional mutations acquired later in disease. This includes a clinically meaningful mutation in RB1 which likely contributed to a change in phenotype to neuroendocrine features, prompting a change in management strategies. The RB1 gene is altered in nearly 9% of advanced prostate cancer cases, through deletion, frameshift mutations, and introductions of premature stop codons [22]. Beltran et al. compared advanced prostate neuroendocrine and adenocarcinoma, demonstrating that RB1 alterations are significantly enriched in advanced prostate cancer with neuroendocrine features (70% altered) compared to that with pure adenocarcinoma features (32% altered) [23]. Loss of RB1 function is common in primary small cell cancer of the prostate or lung, and in animal models it promotes development of small cell carcinoma [24, 25].

The clonal relationship among all three specimens suggest that neuroendocrine disease arose from adenocarcinoma, rather than being a coincident, independent clone. In addition, the high frequency of mutations in CRPC tissue that were present at low frequency in treatment-naïve tissue supports the idea that advanced disease, including neuroendocrine disease, arises from subclonal population(s) in the initial specimen. Of note, though CTCs from patients with neuroendocrine prostate cancer are more frequently nonclassical than those with patients with adenocarcinoma (17), the RB1 mutation was identified in classical EpCAM+ CTCs.

Fewer mutations were identified in CTCs than in treatment-naïve or CRPC tissue samples. The significance of this is unclear. It may be that the limited number of CTCs was unable to capture the extensive diversity of clones comprising disease in the tissue. Alternatively, it may be that CTCs represent a limited number of aggressive and clinically relevant clones. The CTCs were not clonal, as evidenced by the presence of branch and leaf mutations. This genetic heterogeneity among CTCs is supported by Massard et al. [26] based on a single genomic alteration, the ERG alteration pattern. The extent to which CTCs represent all the relevant subclones needs to be explored further.

There are several advantages of a pooled CTC strategy over single CTC sequencing, including availability of resources. Our strategy relied on FACS-sorting and whole genome amplification using a commercially available kit, which are readily available to most researchers. We did not require laser capture microdissection or robotic micromanipulation. Disadvantages include applicability limited to patients with a higher burden of CTCs and inability to fully characterize heterogeneity at the cellular level.

While we had a much higher success rate sequencing pooled CTCs than has been reported with single CTC sequencing, only one of three pools provided high quality data. This may have been due in part to our multiple displacement amplification strategy [27]. This was chosen for the low error rate of its polymerase, but it may be less reliable in amplifying the majority of the genome compared to PCR-based methods.

As the number of effective therapies used for treatment of advanced prostate cancer increases, there is an increasing appreciation of the dynamic nature of the genomics of advanced disease in response to therapeutic pressure. We demonstrate here that sequencing pooled CTCs is a feasible, noninvasive, and informative way to evaluate the current molecular features of advanced disease.

## Conclusions

The histology, behavior, and genomics of advanced prostate cancer evolve in response to therapeutic pressure. Pooled CTCs are a feasible, non-invasive way to interrogate the molecular characteristics of advanced prostate cancer.

## Additional files

**Additional file 1.** PCR primer sequences.

**Additional file 2.** High confidence somatic mutations.
